# Mother’s Milk and the Environment: Might Chemical Exposures Impair Lactation?

**DOI:** 10.1289/ehp.125-A17

**Published:** 2017-01-01

**Authors:** Lindsey Konkel

**Affiliations:** Lindsey Konkel is a New Jersey–based journalist who reports on science, health, and the environment.

Most studies on breastfeeding over the past few decades have focused on the advantages of breastfeeding and how to get more women to breastfeed their babies. Relatively few women worldwide meet the World Health Organization’s recommendation that infants breastfeed exclusively for the first 6 months of life, with continued breastfeeding combined with appropriate foods thereafter for 2 years or more^1^—even those who intend to do so at the outset. Many factors influence how long a mother nurses, but new mothers often state a common problem when asked why they quit breastfeeding earlier than they wanted: They can’t produce enough milk.^2^

**Figure:**
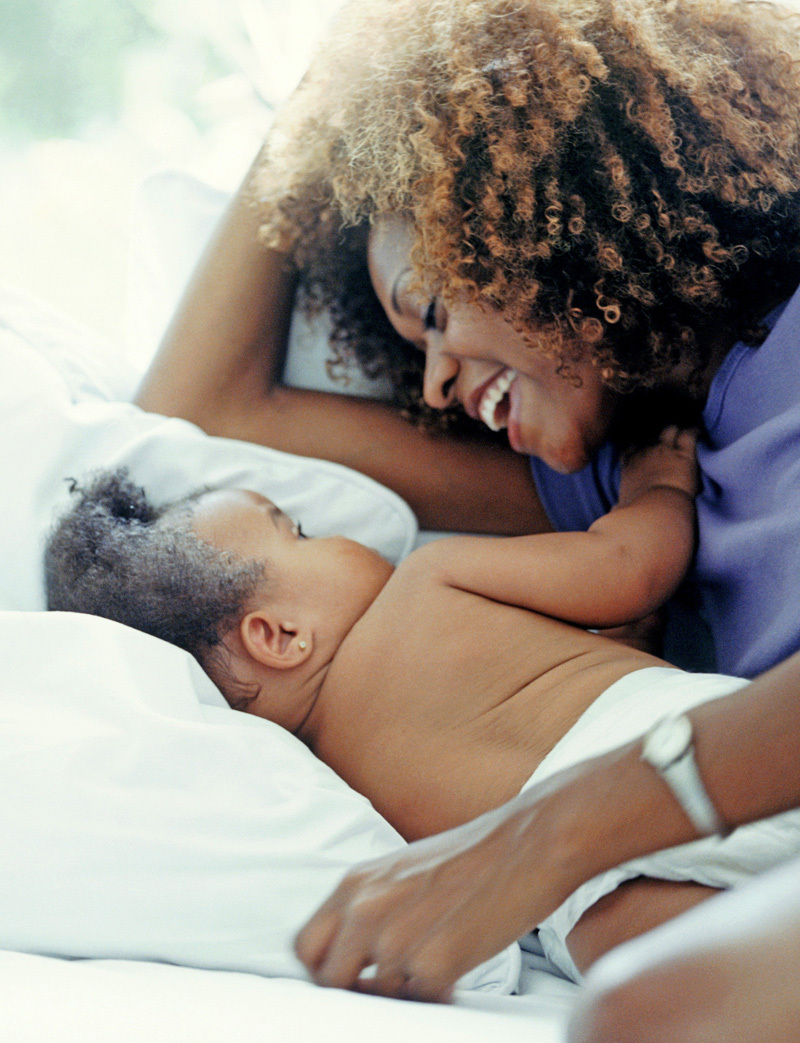
New mothers who quit breastfeeding earlier than they had wanted often chalk it up to not being able to produce enough milk. But a handful of researchers are exploring whether certain environmental exposures may affect some women’s ability to lactate. © Tony Anderson/Getty Images

There is much about lactation that remains unknown. We know a lot about the benefits of breastfeeding for mothers and babies, says Alison Stuebe, an obstetrician/gynecologist at the University of North Carolina School of Medicine, but “we know far less than we should about how the breast functions in lactation and what specific factors might be making it harder than evolution had intended.”

“Very few studies have looked at the factors that may interfere with lactation,” agrees Philippe Grandjean, an epidemiologist at the Harvard School of Public Health and the University of Southern Denmark. Grandjean and others have begun to investigate the potential impacts of chemical exposures on the process of lactation itself. A burgeoning body of research—from rodent toxicology to human epidemiology studies—suggests that certain environmental exposures may impair a mother’s ability to nurse her child.

## Current Thinking on Low Milk Production

Standard lactation support guidelines^3^ are based on the assumption that virtually any woman can breastfeed normally. “The conventional wisdom has been that mothers’ worries about milk supply are due either to their misperception of normal lactation and infant breastfeeding behaviors or to mismanagement of breastfeeding technique, and only on very rare occasions due to an intrinsic inability to make enough milk,” says Laurie Nommsen-Rivers, a perinatal nutrition specialist at the University of Cincinnati College of Allied Health Sciences.

This belief among public health and medical professional is justifiable, says Nommsen-Rivers, because formula companies have a long history of dubious marketing practices that can undermine a woman’s confidence in her ability to nourish her baby without the use of their products.^4^ ^,^ ^5^ Breastfeeding rates began to dwindle worldwide with the advent of infant formula in the 1860s.^4^ They reached an all-time low in the United States in 1971, when fewer than 25% of new mothers initiated breastfeeding at all, and only 5% of mothers were still breastfeeding at 6 months.^6^ Today, largely due to parent education efforts and improved support for lactating mothers, more than 75% of U.S. mothers initiate breastfeeding, and about 20% of U.S. babies are breastfed exclusively through 6 months.^7^

Robust milk production depends on frequent and thorough draining of the breast through suckling or pumping. So a perceived problem with supply can quickly become a real problem once a mother introduces formula and the frequency of breastfeeding declines, especially if formula is offered while a mother is still trying to establish her milk supply.^8^

To complicate matters, a number of key socioeconomic, cultural, and clinical factors also can prevent some women from breastfeeding as long as they would like. Such barriers may include lack of prenatal education about breastfeeding, inadequate lactation support from healthcare providers after delivery, disapproval from spouses or family members, short maternity leave, and few opportunities to pump at work. Lactation support includes breastfeeding-related counseling and education from trained specialists both in the hospital shortly after birth and at home for the first months of the baby’s life.^9^

While an absolute inability to lactate is rare, estimated to occur in less than 2% of mothers,^10^ “we have no idea about the prevalence of women who are lactating less than optimally, who may be unable to exclusively breastfeed a baby no matter how frequently and thoroughly they breastfeed,” says Nommsen-Rivers.

Stuebe’s research suggests that as many 1 in 8 women wean earlier than they want to, despite receiving lactation care.^11^ Yet there are no real tests to measure optimal breast function, she says. When a woman struggles, Stuebe says, “she is often told to try harder.”

## Early Environmental Clues

In 1949 Morton Biskind, a Connecticut physician, treated a pregnant patient with acute DDT poisoning. After the woman gave birth and began to breastfeed, Biskind noted that her symptoms, which included vomiting, abdominal pain, hyper-irritability, and muscle weakness, began to dissipate. When he analyzed her milk, he found it contained exorbitant levels of DDT.^12^

Two years later, researchers examined milk samples from 32 black women at a Washington, DC, hospital.^13^ None of the women worked with pesticides or reported an acute exposure to DDT, yet their milk contained trace amounts of the chemical. The study provided the first scientific evidence that even low-level exposures to environmental chemicals could contaminate human milk.

By 1978 environmental chemicals were known to be widespread contaminants in human milk. Researchers at the National Institute of Environmental Health Sciences initiated the North Carolina Breast Milk and Formula Project. Led by institute scientists Walter Rogan and Beth Gladen, both now retired, the study aimed to gather data on health effects in infants who were exposed via breast milk to polychlorinated biphenyls (PCBs) and to DDT^14^—which by then had been banned.

The researchers studied 858 mother–infant pairs between 1978 and 1982. They collected milk samples at multiple points throughout lactation and estimated concentrations of PCBs and DDE (a breakdown product of DDT) in milk at birth. Then they compared concentrations of these compounds in mother’s milk to outcomes of child health, growth, and development.

While neither PCBs nor DDE were associated with a discernible difference in how much weight the infant gained or the number of doctor visits for illnesses in the first year of life, the researchers did note one surprising result: The median duration of breastfeeding was shorter in mothers with higher estimated levels of DDE in their milk compared with mothers who had lower levels, an association that was not seen with PCBs. They speculated that “DDE may be interfering with the mother’s ability to lactate, possibly because of its estrogenic properties.”^14^

Over the next three decades, these and other researchers tried to replicate the findings of the North Carolina study. Their work turned up mixed results, which are hard to compare due to differences in study design, such as the timing of sample collection.

For example, in a study of women and infants living in a cotton-growing region of northern Mexico where DDT was heavily used, Gladen and Rogan found that women with the lowest DDE levels in their milk shortly after birth nursed for a median of 7.5 months, while mothers with the highest DDE levels nursed for a median of only 3 months.^15^ A study in Michigan estimated serum levels of DDE at the time of pregnancy based on sampling conducted after birth. Among primiparous (first-time) mothers, women with the highest estimated serum levels of DDE at delivery breastfed for a median of 13.0 weeks, compared with a median of 30.3 weeks for women with the lowest levels; among multiparous mothers—women who had given birth previously—these numbers were 13.0 and 21.7 weeks, respectively. These associations were strongest in nonsmokers. ^16^

**Figure:**
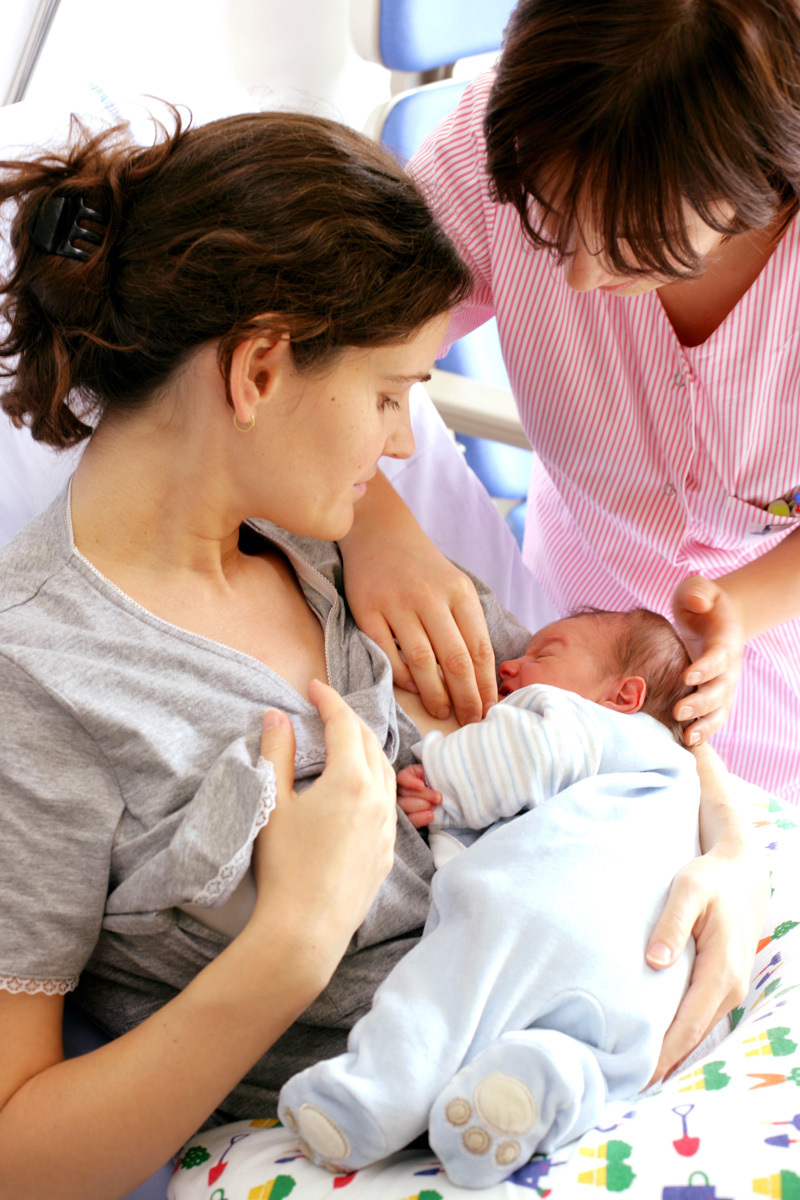
Lactation support includes teaching women techniques to breastfeed and build their milk supply, as well as reassuring them of their ability to nourish their infants. © Phanie/Alamy Stock Photo

**Figure:**
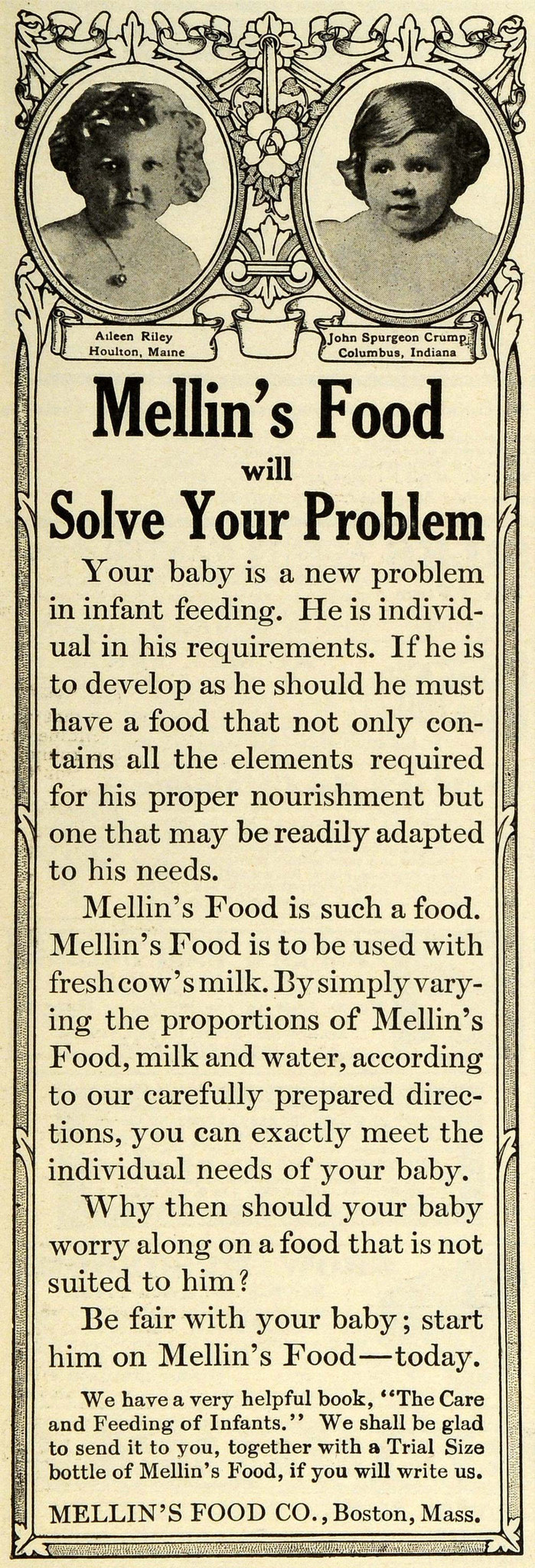
Education and counseling have been important tools in boosting breastfeeding rates, after decades of marketing messages that insinuated breast milk was insufficient to nourish young babies. Mellin’s Food Company

On the other hand, two other studies found no association between DDE/DDT and shortened lactation time. One of the studies looked at serum levels in samples collected from Mexican mothers within a day of birth,^17^ while the other used maternal serum samples collected during the second trimester from Mexican-American women who lived in California’s Salinas Valley, a major agricultural region.^18^

Reverse causality is a major concern in environmental health research, and Amalie Timmermann, a research assistant in the Department of Environmental Medicine at the University of Southern Denmark, points it out as a potential explanation for findings in studies such as these. When a woman lactates, she reduces her own body burden of certain chemicals by transferring them to her nursing child via milk. Therefore, women who have already breastfed or those who breastfeed longer could have lower levels of chemicals in their bodies simply because they have already excreted some of their body burden, not because lower exposures allowed them to lactate normally.

**Figure:**
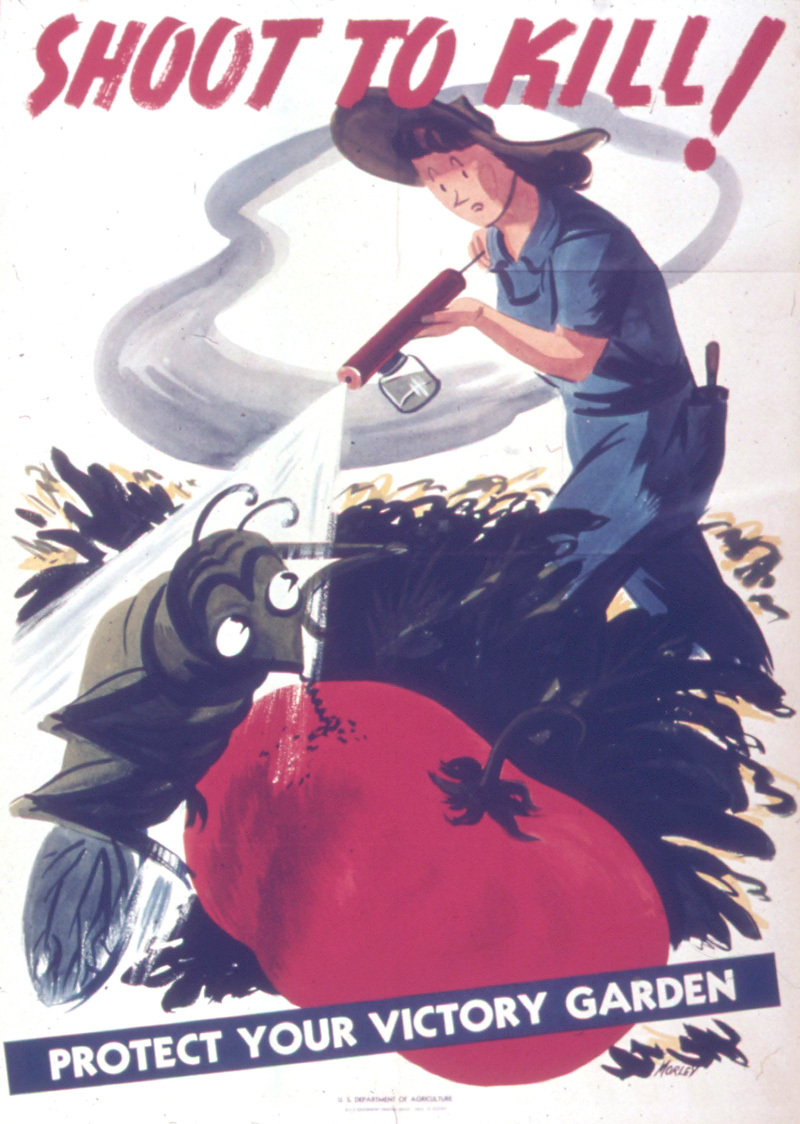
Introduced in the 1940s, DDT was widely used until it was banned for most uses in the 1970s. The 1949 discovery of DDT in breast milk was an early glimpse into the understanding that a mother’s environmental exposures may also affect her baby. Courtesy U.S. National Archives

There are other reasons why reported associations may not actually reveal much about environmental impacts on lactation. For example, Michael Kramer, a perinatal epidemiologist at McGill University in Montreal, points out that investigators are more likely to submit, and journal editors to accept, papers reporting significant associations than those showing no such association—a phenomenon known as publication bias, which can affect observational studies in any field. He also suggests that if a lactating mother were informed of the contaminant levels in her milk, she may be scared to continue breastfeeding or even discouraged from doing so by her healthcare provider.

## New Evidence Emerges

For nearly 30 years Philippe Grandjean has led studies on the effects of marine contaminants on the health of adults and children in the Faroe Islands,^19^ an archipelago in the North Atlantic about halfway between Scotland and Iceland. Among other findings, Grandjean’s team linked postnatal exposure to a group of chemicals called perfluoroalkyl substances (PFASs) with lower immune response to tetanus and diphtheria vaccinations in Faroese children.^20^ PFASs are widely used chemicals that build up in the bodies of humans and other animals. They are present in most humans and are found in breast milk.^21^

Grandjean wanted to know how breastfeeding correlated with some of the child health outcomes his group had observed. “We started to look at breastfeeding as a confounding factor in our studies primarily because it is known to be beneficial to child health,” he says.

Not surprisingly, they found that children who breastfed longer had higher levels of PFASs in their blood. When Grandjean and his team dug a little deeper, they saw that women with the highest PFAS levels in their blood tended to spend less time breastfeeding their babies.^22^

In the study, led by Timmermann—who at the time was pursuing her PhD under Grandjean’s supervision—the researchers estimated the average duration of total breastfeeding (i.e., with or without any other sources of nutrition) in association with serum levels of two PFASs, perfluorooctane sulfonic acid (PFOS) and perfluorooctanoic acid (PFOA). The study combined two cohorts, and serum samples were collected late in pregnancy or approximately 2 weeks after birth. Timmermann and colleagues estimated that breastfeeding was 1.4 months shorter when women’s serum levels of PFOS were doubled and 1.3 months shorter when their levels of PFOA were doubled. The PFAS levels of the Faroese women were comparable to levels reported for pregnant women in Denmark and lower than those reported for a group of U.S. pregnant women.^22^

The findings echoed a 2010 Danish national study, which found associations between higher levels of PFASs in maternal serum samples and shorter duration of breastfeeding.^23^ Yet the Danish study found that this association held true only for multiparous women, and not primiparous mothers. In contrast, in the Faroe Islands study researchers found that exposure to PFASs was associated with shorter breastfeeding duration among both primiparous and multiparous mothers. “This gives us more confidence that the association is not due to reverse causation and is real,” says Timmermann.

Meanwhile, in the United States, another team of researchers pursued a similar line of investigation. Megan Romano, then a postdoctoral research associate at Brown University, looked at 336 women who gave birth at Cincinnati hospitals. Sampling during pregnancy and delivery showed that the women had unusually high serum levels of PFOA—twice the U.S. average—possibly due to the presence of a chemical plant nearby.

**Figure:**
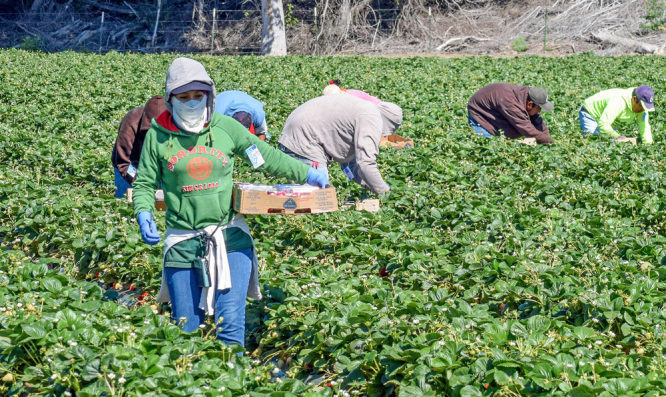
Many studies on DDT exposures and lactation have been conducted with populations of Hispanic women who work on farms or live in agricultural areas. The results from these and other lactation studies have been mixed. © David Litman/Shutterstock.com

Romano and colleagues reported that women with the highest PFOA levels were 77% more likely to stop exclusive breastfeeding by 3 months and 41% more likely to stop by 6 months than women with the lowest levels. More than three-quarters of the Cincinnati women breastfed exclusively for less than 1 month. Roughly two-thirds of the participants were white, and half had attained a college degree or higher. PFAS levels were similar across women’s reported reasons for ending breastfeeding, and the researchers saw no clear differences between primiparous and multiparous mothers.^24^

“You have three studies telling a similar story from different parts of the world. I think it is cause for concern,” says Romano, now an assistant professor at the Geisel School of Medicine at Dartmouth. “Unfortunately, we don’t have a good understanding of what could be going on ‘under the hood’—what biological mechanisms could be driving these associations,” she says. However, some researchers think mammary gland development and maternal metabolism may provide some clues.

## What Might Delay Lactogenesis?

Unlike most organs, which develop mostly *in utero*, the mammary gland develops in stages: before birth, at puberty, and in pregnancy. In that final stage, the milk duct system forms in preparation for lactation and breastfeeding. Changes in hormonal levels dictate breast development at each step.^25^

“We know from animal studies that there are chemicals that can adversely affect breast development and lactation,” says Sue Fenton, leader of the Reproductive Endocrinology Group at the National Toxicology Program, based at the NIEHS. Fenton and colleagues have shown that gestational exposure to PFOA altered the normal formation of the milk duct system in mice.^26^ In a multigenerational mouse follow-up study, they showed that PFOA exposure not only impaired the mouse mothers’ ability to lactate, but also adversely affected prenatal development of the mammary gland in their female offspring. However, neither study was designed to replicate typical human exposures to PFOA, and the doses were much higher than the levels people are typically exposed to in the real world.^27^

It is not clear whether altered development of the mammary gland itself or disruptions in hormonal status are responsible for the negative impacts on lactation that Fenton observed. There is also evidence that PFOA can alter the expression of milk protein genes, which are important for the production of milk.^26^ Fenton further notes that PFASs are only one class of environmental chemicals that may cause concern for lactation.^28^

Poor maternal metabolic health may be another factor that hampers lactation. Multiple national cohort studies have found associations between maternal obesity and shorter duration of breastfeeding,^29^ although the reasons for this relationship are not clear.

Nommsen-Rivers, the perinatal nutrition expert from the University of Cincinnati, initiated breastfeeding studies in both California and Ohio, which were part of the World Health Organization Multicentre Growth Reference Study.^30^ “Our goal was to enable as many women as possible to follow breastfeeding guidelines by providing lactation support,” she says. Yet she was surprised by the high prevalence of delayed lactogenesis—meaning no signs of copious milk production within the first 72 hours of giving birth—in mothers who were striving to breastfeed exclusively.^31^ “I kept thinking, how did our species survive?” she says. “It’s not supposed to be this hard.”

Nommsen-Rivers also observed a cluster of variables, all linked with glucose intolerance, that were significantly associated with delayed lactogenesis. This prompted her to begin investigating the role of insulin in stimulating milk production. Although insulin resistance is a physiologic hallmark of obesity, she says insulin previously was thought to have little, if any, role in lactation.

**Figure:**
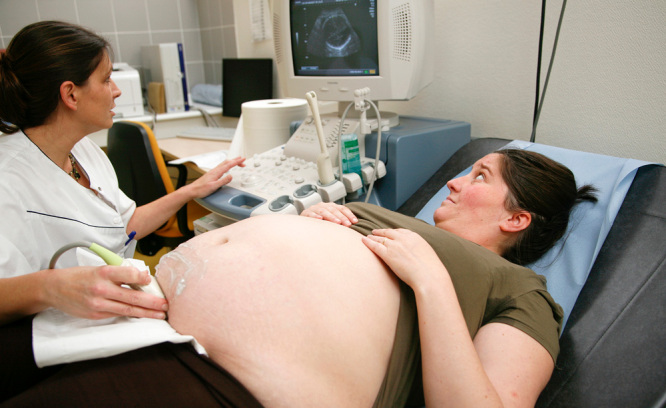
Maternal obesity has been associated with shorter duration of breastfeeding. Although the explanation for this association is still unknown, studies in the past several years have hinted that insulin resistance may be involved in reduced lactation. © BSIP/UIG/Getty Images

She found that measures of glucose intolerance predicted delayed lactogenesis in a group of primiparous Sacramento-area mothers.^10^ In a small group of Cincinnati mothers, she showed that women with weaker insulin response tended to start lactating later than those with good insulin response, with the onset of lactogenesis ranging from 10 to 121 hours postpartum.^32^ When Nommsen-Rivers looked at insulin-sensitive genes in the human mammary gland, she found a potential biomarker linking insulin resistance and difficulty lactating.^33^

She then compared milk production over a 24-hour period in mothers with evidence of insulin resistance versus mothers without. This work showed that women with signs of insulin resistance produced about half as much milk as those without.^34^ It is important to note, she says, that all the women in this study had concerns about their milk production and were already supplementing with formula.

## Many Research Gaps to Fill

Many experts point out that the health benefits of breastfeeding almost always outweigh the costs of exposure to environmental chemicals through breast milk.^35^ Worldwide, breastfeeding has been shown to lower the rate of infant infection, and in developing countries it is strongly protective against death from infection in the first few years of life.^36^ Breastfed babies also are less likely to die of sudden infant death syndrome.^37^ Mothers who breastfeed have a lower risk of breast cancer later in life.^38^

In the developed world, the most important health gains associated with breastfeeding may be cognitive, says Kramer. Some epidemiological evidence suggests that children who are breastfed exclusively during the first several months tend to have higher IQs than those fed formula or a combination of formula and breast milk.^39^ However, there is some debate as to whether breastfeeding actually makes children more intelligent or whether associations between breastfeeding and IQ could be due largely to parents’ intelligence.^40^ Some studies also suggest that breastfed babies have a lower risk of obesity, asthma, and other allergies.^41^

But despite the body of evidence in humans and animals suggesting that ubiquitous environmental exposures may interfere with this highly beneficial process, opportunities to further probe these associations remain spotty at best, say many of the researchers interviewed for this article. Part of the problem is that it is difficult to design breastfeeding studies.

“There are inherent difficulties with studying breastfeeding prospectively in a rigorous manner,” explains Tonse Raju, chief of the Pregnancy and Perinatology Branch at the Eunice Kennedy Shriver National Institute of Child Health and Human Development. For example, researchers could not ethically randomize people such that some were not allowed to breastfeed. As a result, breastfeeding studies may not pass muster with funding committees, says Raju.

In 2011 the U.S. Surgeon General issued “A Call to Action to Support Breastfeeding.”^42^ This document urged more research support for topics related to lactation. In response, Raju helped to initiate the Breastfeeding and Human Lactation Research Scientific Interest Group, a group of scientists organized under the auspices of the National Institutes of Health to identify and discuss research gaps.^43^ “The intention is to increase dialogue across institutions and various funding agencies to stimulate interest in breastfeeding research,” says Raju. He believes there is far more research that needs to be done to understand the barriers to breastfeeding and why some women have difficulties with lactation.
